# Mussel-inspired HA@TA-CS/SA biomimetic 3D printed scaffolds with antibacterial activity for bone repair

**DOI:** 10.3389/fbioe.2023.1193605

**Published:** 2023-05-09

**Authors:** Cheng Ji, Chengcheng Zhang, Zeya Xu, Yan Chen, Yanming Gan, Minghui Zhou, Lan Li, Qinying Duan, Tingting Huang, Jinxin Lin

**Affiliations:** ^1^ Quanzhou Institute of Equipment Manufacturing, Fujian Institute of Research on the Structure of Matter, Chinese Academy of Sciences, Quanzhou, Fujian, China; ^2^ Fujian Science and Technology Innovation Laboratory for Optoelectronic Information of China, Fuzhou, Fujian, China; ^3^ College of Chemistry and Materials Science, Fujian Normal University, Fuzhou, China; ^4^ Fujian College, University of Chinese Academy of Sciences, Fuzhou, China; ^5^ Fujian Medical University, Fuzhou, China

**Keywords:** 3D printing, curcumin, scaffolds, DMON, antibacterial

## Abstract

Bacterial infection is a major challenge that could threaten the patient’s life in repairing bone defects with implant materials. Developing functional scaffolds with an intelligent antibacterial function that can be used for bone repair is very important. We constructed a drug delivery (HA@TA-CS/SA) scaffold with curcumin-loaded dendritic mesoporous organic silica nanoparticles (DMON@Cur) via 3D printing for antibacterial bone repair. Inspired by the adhesion mechanism of mussels, the HA@TA-CS/SA scaffold of hydroxyapatite (HA) and chitosan (CS) is bridged by tannic acid (TA), which in turn binds sodium alginate (SA) using electrostatic interactions. The results showed that the HA@TA-CS/SA composite scaffold had better mechanical properties compared with recent literature data, reaching 68.09 MPa. It displayed excellent degradation and mineralization capabilities with strong biocompatibility *in vitro*. Furthermore, the antibacterial test results indicated that the curcumin-loaded scaffold inhibited *S.aureus* and *E.coli* with 99.99% and 96.56% effectiveness, respectively. These findings show that 3D printed curcumin-loaded HA@TA-CS/SA scaffold has considerable promise for bone tissue engineering.

## 1 Introduction

Bone defects that exceed critical size deficiencies require surgical intervention to use bone grafts to repair the damaged tissue (Boos et al., 2010). Traditional treatment methods include autologous bone grafting and allogeneic bone grafting by obtaining bone tissue from the patient or donor and then implanting it into the bone defect site. Among them, autologous bone graft has complete histocompatibility without immune reaction and is also known as the gold standard of clinical treatment. However, conventional treatment is limited by the lack of bone donors, inflammation, infection, chronic pain at the donor site, and long-term medical care (Koushik et al., 2023; Mirkhalaf et al., 2023). In addition to the traditional treatment methods, Bioactive scaffolds have emerged as an alternative strategy for treating bone defects since interconnected pore structures allow space for inward tissue growth and oxygen and nutrient delivery (Zhang et al., 2019; Jodati et al., 2020). Moreover, bioactive scaffolds avoid the drawbacks of traditional bone repair procedures, such as lack of bone cells and immune rejection, which can considerably reduce the patient’s suffering from bone diseases (O'Brien, 2011; Delloye et al., 2007). The ideal scaffold is also required to have superior osteoinductivity, matching the new bone production rate’s degradation rate, mechanical strength compatible with the original bone, etc., (Lee et al., 2022; Shi et al., 2022). Generally, bone tissue engineering scaffolds are available in four categories: natural polymers, synthetic polymers, bioceramic materials, and composite biomaterials (Boos et al., 2010; Lee et al., 2022; O'Brien, 2011; Zhang et al., 2019). Organic/inorganic composites as bone scaffolds have been the subject of extensive research to imitate the structure of natural bone, which comprises 60–65 wt% inorganic hydroxyapatite (HA) embedded in an organic collagen matrix (Olszta et al., 2007; Shi et al., 2022). There are various ways to make these bone scaffolds, but 3D printing has shown excellent capabilities in fabricating scaffold materials with complex shapes and individual customization (Zhang et al., 2019). 3D printing has structural design advantages and can couple functional designs to achieve structural and functional integration (Brunello et al., 2016; Zafar et al., 2019; Zhang et al., 2019; Wang et al., 2020; Yadav et al., 2021; MacDonald et al., 2022). Our previous research reported that 3D-printed La^3+^ ions-doped OCP/PLA scaffolds could promote osteogenic differentiation of BMSCs and accelerate bone defect healing *in vivo* (Xu et al., 2022). Nevertheless, synthetic polymers' hydrophobicity frequently causes cell adhesion problems, which limits their use. Natural polymers (like chitosan, sodium alginate, collagen, etc.) are similar to the extracellular matrix (ECM) structure. They have outstanding biocompatibility, cell adhesion, and cell growth-promoting qualities (Donnaloja et al., 2020; Guo et al., 2021; Ressler, 2022; Ye et al., 2022). Because of this, researchers are paying more and more attention to them. Mussels, marine organisms, can adhere firmly to a range of non-specific surfaces by secreting protein fibres containing the catechol fraction (Barros et al., 2021; Radulescu et al., 2022). In recent years, there had been remarkable success in the functionalization of mussel-inspired material surfaces (Lee et al., 2007; Mehdizadeh et al., 2012; Guo et al., 2016; Guo et al., 2017; Guo et al., 2018; Lu et al., 2020). Tannins with catechol moieties had been shown to adhere strongly to inorganic surfaces, enhancing the interfacial forces of the complexes (Guo et al., 2020; Yan et al., 2020; Yu et al., 2023).

In clinical, however, implant-related infection is the major concern for implant failure. *Staphylococcus aureus* is the most common pathogen of infections (Lew and Waldvogel, 2004). It can lead to the failure of bone repair and even the death of the patient. Antibiotics was the most effective treatment strategy for bone implant infections, but systemic delivery may cause significant adverse effects and bacterial medication resistance (Ribeiro et al., 2012; Fernandes et al., 2017; Filipovic et al., 2020). Developing and manufacturing bone scaffolds with antibacterial activity is necessary. For instance, Lei et al. reported a ZIF-8-loaded vancomycin bone scaffold with an inhibition efficiency of about 93.5% against *S.aureus*, which exhibited a faster VAN release rate in a weakly acidic solution (Han et al., 2022). In addition, many other types of drugs are used for the antimicrobial functionalization of bone scaffolds, such as doxycycline (Bakhsheshi-Rad et al., 2018; Wang et al., 2021a), tannic acid(TA) (Zhang et al., 2022a; Liao et al., 2022; Zhou et al., 2022), etc. Turmeric is used in Traditional Chinese Medicine (TCM) to treat and prevent various diseases such as osteoarthritis, cancer, stomach ulcers, etc., (Chen et al., 2022). Turmeric has more than 300 bioactive components, including curcumin(Cur), a class of natural orange-yellow polyphenolic compounds found in turmeric (Iweala et al., 2023). Extensive studies had shown it has good antioxidant, anti-inflammatory, antibacterial, and antiviral activities (Benameur et al., 2022; Benameur et al., 2023; Nasiry and Khalatbary, 2023). But curcumin has very low solubility in an aqueous solution and is easily degraded, which limits its clinical application (Hamilton and Gilbert, 2023; Jiang et al., 2023). Therefore, a suitable curcumin carrier is important to improve its bioavailability. There had been several drug release studies for curcumin, such as cellulose nanocrystals containing curcumin for an antimicrobial delivery system for diabetic wound excipients, and the drug can be released stable for 36 h (Ou et al., 2018; Tong et al., 2018). Studies have shown that porous materials combined with low-solubility drugs can significantly improve drug solubility (Kong et al., 2016; Pezzini et al., 2016; Riachy et al., 2016). Dendritic mesoporous organic silica nanoparticles (DMON) can significantly enhance low-solubility drug delivery and release due to their large surface area, high porosity, excellent biocompatibility, and biodegradability (Wang et al., 2021b). Tannin acid is a class of natural water-soluble polyphenol dendrimers with antioxidant and antibacterial properties. Studies have shown that redox-active antioxidants can improve curcumin’s chemical stability and biological activity (Nimiya et al., 2016). In addition, Tannin acid is an efficient precursor for surface treatment, capable of layer-by-layer assembly and deposition with polymer molecules through non-covalent bonds (Zhang et al., 2022a; Liao et al., 2022; Zhou et al., 2022). As a strong adhesive, tannic acid can also easily adhere to the surface of hydroxyapatite (Guo et al., 2020). Therefore, tannins are increasingly used in antibacterial bone scaffolds.

**FIGURE 1:**
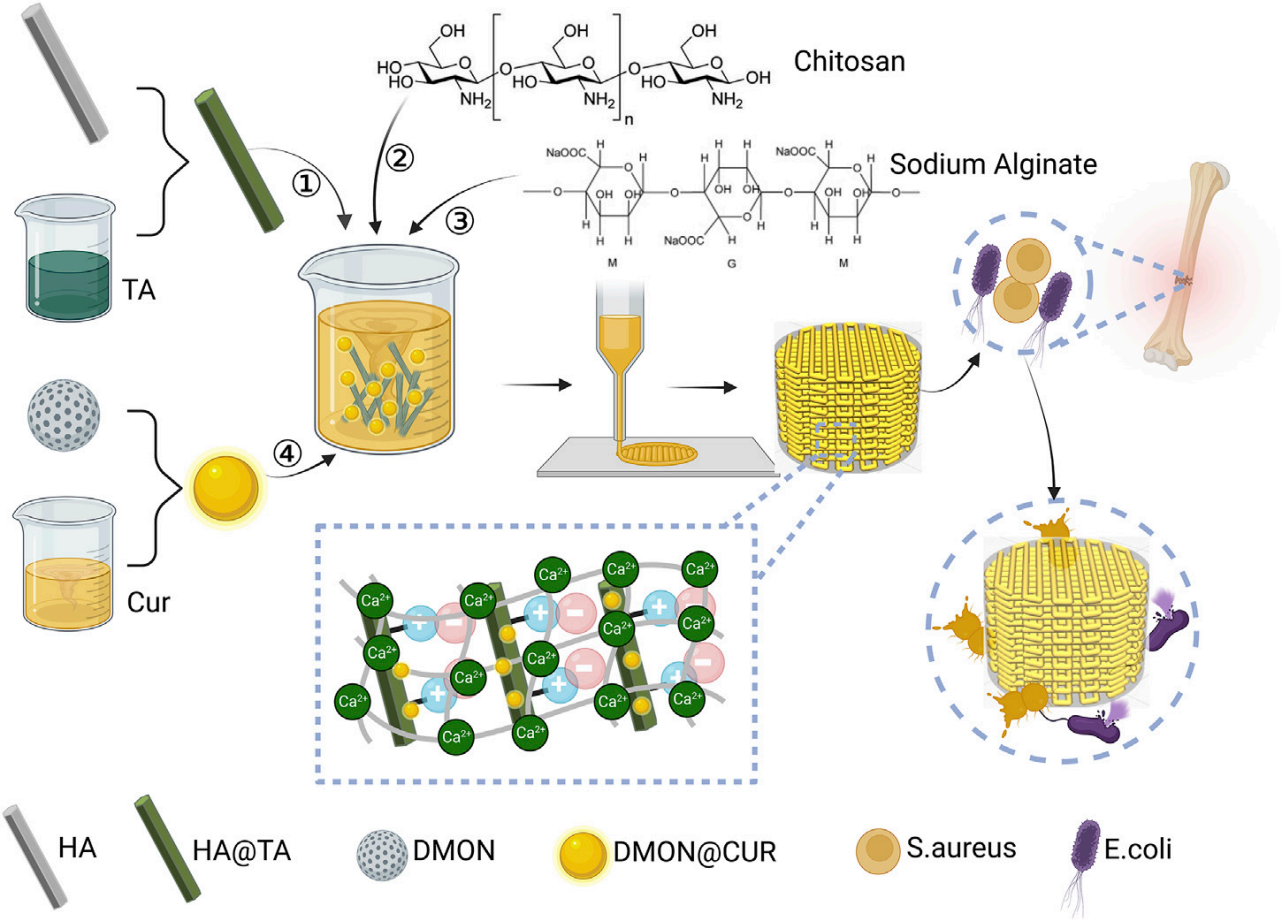
Design ideas and preparation of curcumin-loaded HA@TA-CS/SA scaffolds created with BioRender.com.

Inspired by the adhesion mechanism of mussels, this study prepared HA@TA powder by adhering TA to HA surface. TA was then used as a “bridge” to connect HA and CS, compounded with SA using electrostatic interactions, and doped with DMON@Cur nanoparticles of different mass fractions to produce curcumin-loaded HA@TA-CS/SA scaffolds by 3D printing. As shown in Figure 1, this bionic scaffold had a fully interconnected porous structure that provides a site for tissue ingrowth and nutrient transport, and a rough surface microstructure that promotes cellular and bacterial adhesion. All components of the scaffold are biologically active and degradable compared to other biomaterials, with curcumin providing significant antibacterial properties to the scaffold. This bionic antimicrobial scaffold has great potential in reducing bacterial infections during bone defect repair.

## 2 Experimental section

### 2.1 Materials

Analytical reagent diammonium hydrogen phosphate ((NH_4_)_2_HPO_4_), sodium alginate (SA.), chitosan (CS. high viscosity, >400 mPa.s), and glycolic acid (GA, 70% aqueous) were purchased from Shanghai Aladdin Bio-Chem Technology Co., Ltd (China). Acetamide (AA), hexadecyl trimethyl ammonium bromide (CTAB), bis(3-(triethoxysilyl)propyl) tetrasulfide (BTES), tetraethoxy-silane (TEOS), curcumin(Cur) and tannic acid (TA) were procured from Macklin Biochemical Technol-ogy Co., Ltd (China). Calcium chloride dehydrate (CaCl_2_·2H_2_O, 99%), Tris(hydroxymethyl)amino-methane (C_4_H_11_NO_3_,99.8%), and calciumnitrate tetrahydrate (Ca(NO_3_)_2_·4H_2_O analytically pure) were purchased from Sinopharm Chemical Reagent Co., Ltd (China). All chemicals were used as received without further purification.

### 2.2 Preparation and characterization of powder

#### 2.2.1 Preparation of DMON and DMON@Cur

The DMON synthesis was based on the previously published technique with certain modifications (Dong et al., 2022). The following procedures were carried out in the dark to produce DMON@Cur powder. Ultrasonically dispersing 0.1 g of curcumin and 0.1 g of DMON powder in 50 mL of PBS-Tween 80 (0.09 wt%) took 10 min. This was followed by magnetic stirring at 37°C and 500 rpm for 12 h. The bottom precipitated pellet was then dried under a vacuum at 37°C after high-speed centrifugation. The process’s supernatant was collected, and its UV absorbance at 425 nm was measured using a UV-6100 UV spectrophotometer (Mwere dissolved in 200 mL of Acetamide (AA) solution (1 mol/L) aAPADA) to determine the encapsulation efficiency and loading. A standard calibration curve of Cur in PBS-Tween 80 (0.09 wt%) was used to determine how much Cur was in the supernatant. Eq. 1, 2 were used to determine encapsulation efficiency and loading capacity.

$$Encapsulation\;efficiency\;\left(\%\right)=\frac{Loaded\;amount\;of\;CUR}{Total\;amount\;of\;CUR}$$ (1)

$$Loading\;capacity\;\left(\%\right)=\frac{Loaded\;amount\;of\;CUR}{Total\;amount\;of\;DMON}$$ (2)

#### 2.2.2 Synthesis of long rod-shaped hydroxyapatite modified with tannic acid

Hydroxyapatite (HAp, Ca_10_(PO_4_)_6_ (OH) _2_) powder samples were prepared via the pH-adjusting agents-assisted hydrothermal synthesis. In a typical experiment (Zhang and Darvell, 2010), 0.025 mol of Ca(NO_3_)_2_·4H_2_O and (NH_4_)_2_HPO_4_ were dissolved in 200 mL of Acetamide (AA) solution (1 mol/L) and then mixed slowly at a Ca/P ratio of 1.67. Then the pH was adjusted to 3.0 with 0.1 mol/L of HNO_3_. The resulting reaction system was reacted hydrothermally at 180°C for 6 h and then filtered and washed to obtain pure HA powder.

To synthesize HA@TA powder (Guo et al., 2020), first dissolved an appropriate amount of Tris(hydroxymethyl)aminomethane in deionized water and adjusted the pH to 9.0 with 1 mol/L hydrochloric acid to form 50 mM Tris-HCl solution. Then, 5 g of tannic acid was added to 100 mL of Tris-HCl solution and stirred until clear, and an equal mass of HA powder was dispersed in 150 mL of Tris-HCl solution. The above solutions were mixed and sealed at room temperature under mechanical stirring for 24 h. Finally, the above yellow-green suspension was centrifuged at 4500 rpm for 10 min, washed 3 times with deionized water, and dried in an oven at 60°C for 6 h.

#### 2.2.3 Characterization of powder

Field emission scanning electron microscopy (FESEM; SU8010, Hitachi) and transmission electron microscopy (TEM; TecnaiG2F20S-TWIN, FEI) analyses were used to examine the microstructure and distribution of the synthesized HA and DMON. The products' corresponding elemental mapping and energy-dispersive X-ray spectra (EDS) were obtained from the TEM. The surface area and pore size of DMON were measured on a Micromeritics TristarII 3020 system using an N_2_ adsorption-desorption isotherm. The samples were degassed under vacuum at 120 °C for 24 h before testing. The crystalline nature of HA, HA@TA powders were identified by X-ray diffraction (XRD, Miniflex600, Rigaku) using Cu Kα radiation at 40 kV and 15 mA current. The 2θ angles were scanned from 10° to 65° at a scan rate of 2°/min. The chemical and functional characteristics of HA, HA@TA, DMON, and DMON@Cur powders were examined by FTIR spectroscopy (Lambda 950, Perkin Elmer). Raman spectrophotometer (LabRAM HR, HORIBA) was also used to characterize the KBr integrated samples, using an Nd: YAG laser with a wavelength of 633 nm, a displacement range of 400–4000 cm^-1,^ and a resolution of 2 cm^−1^. The surface potential of HA, HA@TA powders identified with the BI-200SM dynamic light scattering particle size analyzer.

### 2.3 Preparation of scaffolds

Table 1 lists the names of all the scaffolds prepared in this work.

**TABLE 1:** The names and codes of the prepared scaffolds.

Scaffold name	Code	DMON@Cur (wt%)
Hydroxyapatite-Sodium alginate	HA-SA	0
Hydroxyapatite@ Tannin-Sodium alginate	HA@TA-SA	0
Hydroxyapatite@ Tannin-Chitosan/Sodium alginate	HA@TA-CS/SA	0
Hydroxyapatite@ Tannin-Chitosan/Sodium alginate-3 wt% DMON curcumin	HA@TA-CS/SA-3 wt% DMON	3
Hydroxyapatite@ Tannin-Chitosan/Sodium alginate-6 wt% DMON curcumin	HA@TA-CS/SA-6 wt% DMON	6
Hydroxyapatite@ Tannin-Chitosan/Sodium alginate-9 wt% DMON curcumin	HA@TA-CS/SA-9 wt% DMON	9

#### 2.3.1 Preparation of HA@TA-CS/SA scaffolds and 3D printing

The slurry of the HA@TA-CS/SA scaffold for printing requires an inorganic-organic mass ratio mimicking natural human bone of 6:4, which is prepared by the *in situ* compounding method. Specifically, 1 g of CS was dissolved in 200 mL of ethanolic acid (2 wt%) to prepare a 5 g/L solution of CS. 2 g of the prepared HA@TA powder was dispersed in 150 mL of deionized water and stirred continuously with a magnetic stirrer for 20 min to achieve uniform dispersion. 53.33 mL of CS solution was added to the above solution by high-speed magnetic stirring for 30 min, and the pH of the solution was adjusted to 7.6 by adding 1% sodium hydroxide solution dropwise. Some of the above-stirred slurries were dried in the oven at 60 °C to obtain HA@TA-CS powder, which was then used for subsequent characterization tests. Then, 1.07 g of SA powder was weighed and slowly added to the above solution under high-speed mechanical stirring so that the mass ratio of CS to SA was 2:8. Stirring was continued until the SA was dissolved entirely so that the slurry was well mixed. Some of the above-stirred slurries were dried in an oven at 60 °C to obtain HA@TA-CS/SA powder, which was used for subsequent characterization tests. The resulting solution was frozen in a refrigerator at −20 °C for 12 h and then freeze-dried for 48 h until complete. The freeze-dried "foamy" solid was mixed with deionized water in a ratio of 1:3 by mass and stirred with a glass rod until it was in the form of a slurry. The prepared slurry is then loaded into a 50cc cylinder and waited for printing.

The cylindrical models of Φ15 × 5 mm and Φ15 × 30 mm were constructed using SolidWorks2020 modeling software, saved as stl format files, imported into the model slices using CurA slicing software, set specific process parameters: layer thickness 0.6 mm, filling density 50%, filling path serrated, filling direction 90°, printing speed 25 mm/min. After that, the Gcode file is generated, and the porous scaffold is printed layer by layer by an extrusion 3D printer whose needle diameter is 0.6 mm under the control of a computer program. After printing, the scaffolds were crosslinked in 10 wt% CaCl_2_ solution for 5 h, then washed three times with deionized water to remove the CaCl_2_ solution from the surface and dried in an oven at 37°C for 24 h.

#### 2.3.2 Characterization of HA@TA-CS/SA scaffolds

The crystalline nature of HA@TA-CS and HA@TA-CS/SA powders were identified by X-ray diffraction (XRD, Miniflex600, Rigaku). The chemical and functional characteristics of HA@TA-CS and HA@TA-CS/SA powders were examined by FTIR spectroscopy (Lambda 950, Perkin Elmer) and Raman spectrophotometer (LabRAM HR, HORIBA) to explore their interactions in the scaffold composites. Elemental and bond composition of HA@TA-CS/SA powders used by x-ray photoelectron spectroscopy (XPS). The surface potential of HA@TA-CS and HA@TA-CS/SA powders were identified with the BI-200SM dynamic light scattering particle size analyzer.

The rheology and viscoelasticity of the printed slurry were tested at 25°C using a rheometer (TA Instruments, DHR-2). The rheology of the slurry was tested in flow scan mode using a 25 mm diameter 5.805° conical-plate-shaped rotor with a measurement gap of 150 μm. The viscoelasticity of the slurry was tested using the same rotor in amplitude scan mode at 1 Hz and with a measurement gap of 150 μm. FESEM carried out the microscopic morphology of the prepared HA@TA-CS/SA scaffold at 5 kV after gold sputter coating.

#### 2.3.3 Preparation of curcumin-loaded HA@TA-CS/SA scaffolds

DMON@Cur nanoparticles with varying mass ratios (3.0, 6.0, and 9.0 wt%) were added to the above HA@TA-CS/SA slurry to make the scaffolds antibacterial. It is worth noting that the remainder of the preparation procedure was identical. Portions of the above-stirred slurries were baked in a 60 °C oven to produce curcumin-loaded HA@TA-CS/SA powder for future characterization testing.

#### 2.3.4 Characterization of curcumin-loaded HA@TA-CS/SA scaffolds

The chemical and functional characteristics of curcumin-loaded HA@TA-CS/SA powder were examined by Fourier transform infrared spectroscopy (Lambda 950, Perkin Elmer).

The rheology and viscoelasticity of the printed slurry were tested at 25°C using a rheometer (TA Instruments, DHR-2). The rheology of the slurry was tested in flow scan mode using a 25 mm diameter 5.805° conical-plate-shaped rotor with a measurement gap of 150 μm. The viscoelasticity of the slurry was tested using the same rotor in amplitude scan mode at 1 Hz and with a measurement gap of 150 μm. FESEM carried out the microscopic morphology of the prepared curcumin-loaded HA@TA-CS/SA scaffolds at 5 kV after gold sputter coating. The thermal stability of the scaffold’s powder was measured using a comprehensive thermal analyzer (STA449-F3, Netzsch), with a heating rate of 10 k/min and a measurement interval of 30°C–900°C.

### 2.4 Compression testing

For the compressive strength tests, two more control scaffolds (HA-SA and HA@TA-SA) were prepared using the controlled variable method to see how the different parts of the HA@TA-CS/SA scaffolds worked together. The HA-SA and HA@TA-SA scaffolds were fabricated using the same fabrication process, and both had an organic/inorganic component ratio of 4:6. The 3D-printed HA-SA, HA@TA-SA, HA@TA-CS/SA, and curcumin-loaded HA@TA-CS/SA scaffolds were all Φ15 × 30 mm in size. The compressive stress and Young’s modulus of the porous scaffolds were measured using a universal testing machine (CMT4304, SANS).

### 2.5 Immersion tests

The *in vitro* degradation test of the curcumin-loaded HA@TA-CS/SA scaffolds was conducted in PBS solution (pH = 7.4) at 37°C to evaluate the weight loss. The exact weight of the scaffold (Wi) was measured, and then the scaffold was put into a beaker with PBS. Under the aforementioned conditions, the beakers were sealed entirely and kept at 37°C for 14 days. At set times, samples were taken out, washed, freeze-dried, and weighed (Wf), and the shape of the degraded scaffold was examined using a FESEM microscope. Eq. 3 was used to determine the percentage degradation rate (DR):

$$DR\;\left(\%\right)=\frac{Wi-Wf}{Wi}$$ (3)

### 2.6 Curcumin release studies

To evaluate the drug release pattern of curcumin-loaded scaffolds in normal and inflammatory environments, scaffolds containing different concentrations (3.0,6.0,9.0 wt%) of DMON@Cur particles were placed into dialysis bags (30 kDa, Solarbio, China). Meanwhile, A set of HA@TA-CA/SA-loaded curcumin scaffolds without DMON nanoparticles, in which the curcumin loading concentration was 9 wt%, was also prepared and put into the dialysis bag as a comparative test. The dialysis bags were placed in 50 mL of PBS-Tween80 (0.9 wt%, 100 rpm/min) solution at pH 5.3 and pH 7.4, respectively, as a release medium without ambient light. At defined time intervals, 3 mL of the solution was removed and replaced with a new PBS-Tween80 solution. Samples obtained from the release medium were characterized using a UV spectrophotometer at 425 nm to determine the concentration of released Cur.

### 2.7 Cytocompatibility assay

Bone marrow mesenchymal stem cells (BMSCs) of 3-5 generations were used for the study of *in vitro* cellular experiments. Primary rat bone marrow MSCs were isolated and cultured following previously reported procedures. BMSCs were cultured in culture flasks and maintained at 37°C as well as 5% CO_2_ in a cell culture chamber (SCO5W-2, Shellab, United States). Cells were cultured with DMEM/F12 medium containing 10% fetal bovine serum, 1% streptomycin-penicillin double antibody and 1% glutamine, renewed every 2 days. All curcumin-loaded HA@TA-CS/SA scaffold extracts were prepared according to ISO 10993–12 2017. The curcumin-loaded HA@TA-CS/SA scaffolds were immersed in ethanol (75 v/v%) for 2 h, then rinsed with sterile PBS and immersed in the medium at 10 ± 0 mg/mL (same as cell culture medium) and incubated on a shaker at 37°C. The medium was collected, renewed every other day with a sterile syringe, and filtered through 0.22 µm. The filtrate was collected in a 15 mL centrifuge tube and stored in a refrigerator at 4°C for later use.

The cell viability of the curcumin-loaded HA@TA-CS/SA scaffolds was measured by the Cell Counting Kit-8 (CCK-8) assay. In a typical experiment, BMSCs were incubated with extracts at a density of 8 × 104 cells/cm^2^ in 96-well plates. 10 μL CCK-8 solution was added to each well at different time points (1, 3, and 5 days). After 1 h of incubation, each well’s optical density (OD) was measured at 450 nm using an enzyme marker to quantify cell proliferation.

After 1, 3, and 5 days of incubation, bone marrow BMSCs were stained with a calcein-AM/PI double staining kit (Dalian Meilun Biotechnology Co., Ltd.) The fluorescence images of bone marrow MSCs obtained by inverted fluorescence microscopy.

The results of osteogenic differentiation were observed under the microscope after incubation of the three generations of BMSCs cells with the infiltrates of four curcumin-loaded scaffolds for 7 days using alkaline phosphatase staining. ALP activity was then quantified using an alkaline phosphatase assay kit (p0321, Beyotime).

### 2.8 Antibacterial test

With the inhibition circle method, the inhibition ability of the curcumin-loaded HA@TA-CS/SA scaffold against *E.coli* and *S.aureus* was measured. The slant strains of *E.coli* and *S.aureus* used in the experiment were bought from the Shanghai Conservation Biotechnology Centre. Inoculation loops put *E.coli* and *S.aureus* on nutrient agar plates. The plates were then incubated at 90% humidity and 37°C for 20 h until the bacteria entered the exponential growth phase. A small number of bacteria entering the exponential growth phase was picked up with an inoculation loop, dissolved in PBS solution, and diluted to 1 × 108 cfu/mL using the medium diffusion method.

Bacterial inhibition loop experiment using *E.coli* and *S.aureus*: Dilute the above 1 × 108 cfu/mL *E.coli* and *S.aureus* bacterial solution 100 times with PBS solution, use a pipette to take 100 μL of the diluted bacterial solution on an agar plate, spread it evenly with a spreader, allow the bacterial solution to dry and then take a Φ15 × 5 mm size curcumin-loaded HA@TA-CS/SA (0,3,6, and 9 wt%) porous scaffolds were evenly placed in the Petri dishes and incubated upside down in a constant temperature incubator at 37°C for 24 h before comparing the size of the inhibition rings.

The bactericidal rate test is an important measure of whether the scaffold could kill the bacteria in contact with it in the surrounding environment. The above 1 × 108 cfu/mL *Staphylococcus aureus* and *Escherichia coli* solutions were diluted 10 times to 105 cfu/mL in PBS solution. Curcumin-loaded HA@TA-CS/SA (0,3.0,6.0 and 9.0 wt%) scaffolds were placed in a bacterial suspension at a 105 cfu/mL concentration rate of 1 g/100 mL and placed in a 37°C incubator. Plates were counted at 24 h of incubation at 10-fold dilution, each group was repeated three times, and the bactericidal rate was calculated. Bactericidal rates were assessed using Eq. 4:

$$Bactericidal\;rate=\frac{{colony\;count}_{blank}-c{olony\;count}_{sample}}{{colony\;count}_{blank}}$$ (4)

The bacteria were diluted with PBS buffer until the absorbance OD value was 0.1 and then co-cultured with the scaffolds for 12 h. Using the BCA kit, protein concentration was measured.

## 3 Results and discussion

### 3.1 Characterization of DMON@Cur

**FIGURE 2:**
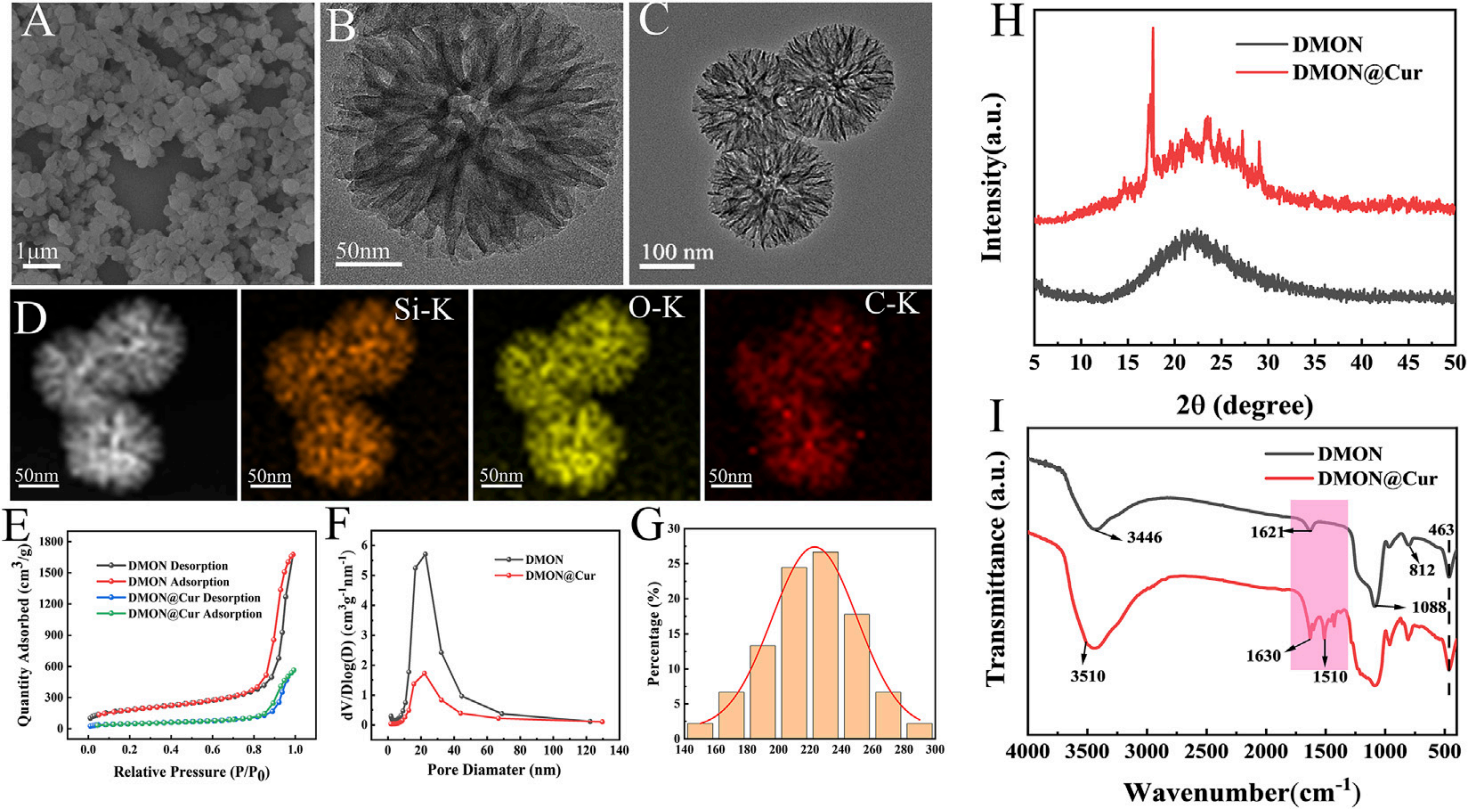
**(A)** SEM micrographs of DMON. **(B)** TEM micrographs of DMON. **(C)** TEM micrographs of DMON@Cur. **(D)** Elemental mappings. **(E)** Nitrogen absorption−desorption isotherm. **(F)** the corresponding pore-size distribution of DMON and DMON@Cur. **(G)** Size distribution of DMON@Cur **(H)** XRD of DMON and DMON@Cur. **(I)** FTIR of DMON and DMON@Cur.

FESEM analysis showed that the organic dendritic mesoporous silica microspheres (DMON) appeared as uniformly sized spherical particles with fullness (Figure 2A). Meanwhile, TEM analysis (Figures 2B, C) revealed the fine surface structure of DMON, highlighting the presence of distinct dendritic mesopores. Mappings (Figure 2D) showed that element C was uniformly distributed on the microspheres' surface and internal pores, proving that curcumin had been loaded onto the DMON microspheres. The specific surface area and pore size distribution of the prepared DMON and DMON@Cur nanoparticles were measured using N_2_ adsorption-desorption isotherms (Figures 2E, F). The N_2_ adsorption-desorption isotherms of both nanoparticles could be classified as type IV, indicating a mesoporous structure(Yao et al., 2020). The specific surface area rapidly reduced after total absorption (from 632.6 m^2^/g to 170.8 m^2^/g). Furthermore, the pore size of DMON was 17.5 nm, which dropped to 15.6 nm in DMON@Cur, suggesting the successful production of Cur-loaded DMON nanosystems. The average size of DMON@Cur particles was approximately 228 ± 5 nm (Figure 2G). The encapsulation efficiency and drug loading capacity of DMON were 73.81% and 70.25%, respectively, calculated by Eqs 1, 2. X-ray diffraction (Figure 2H) and FTIR (Figure 2I) showed the DMON and DMON@Cur structural characteristics spectroscopy. As shown in the XRD patterns (Figure 2H), characteristic diffraction peaks of amorphous SiO_2_ within 15°–30° for mesoporous silica and the main diffraction peaks of curcumin appeared at 2θ = 17–28°(Ou et al., 2018; Oboudatian and Safaei-Ghomi, 2022). The FTIR (Figure 2I) showed the typical peaks of the DMON could be observed in the region of 1092–1150 cm^−1^, reflecting the Si–O–Si asymmetric stretching vibration, Si–O–Si peak was observed at 812 cm^−1^, which represented the symmetric stretching vibration. Moreover, the peaks at 463 cm^−1^, 1621 cm^−1^, and 3446 cm^−1^ correspond to the Si–O bending vibration, the O–H bending vibration, and the stretching vibration, respectively(Oboudatian and Safaei-Ghomi, 2022). The main characteristic peaks of curcumin were the unsaturated carbonyl vibrations at 1630 cm^−1^, the hydroxyl vibrations at 3510 cm^−1^, and the benzene ring peaks at 1600 cm^−1^ and 1510 cm^−1^ (Benassi et al., 2008). It was found that Cur and DMON interacted by hydrogen bonding. All the above results showed that the synthesized DMON nanoparticles could bind well with curcumin through hydrogen bonding.

### 3.2 Characterization of scaffolds

**FIGURE 3:**
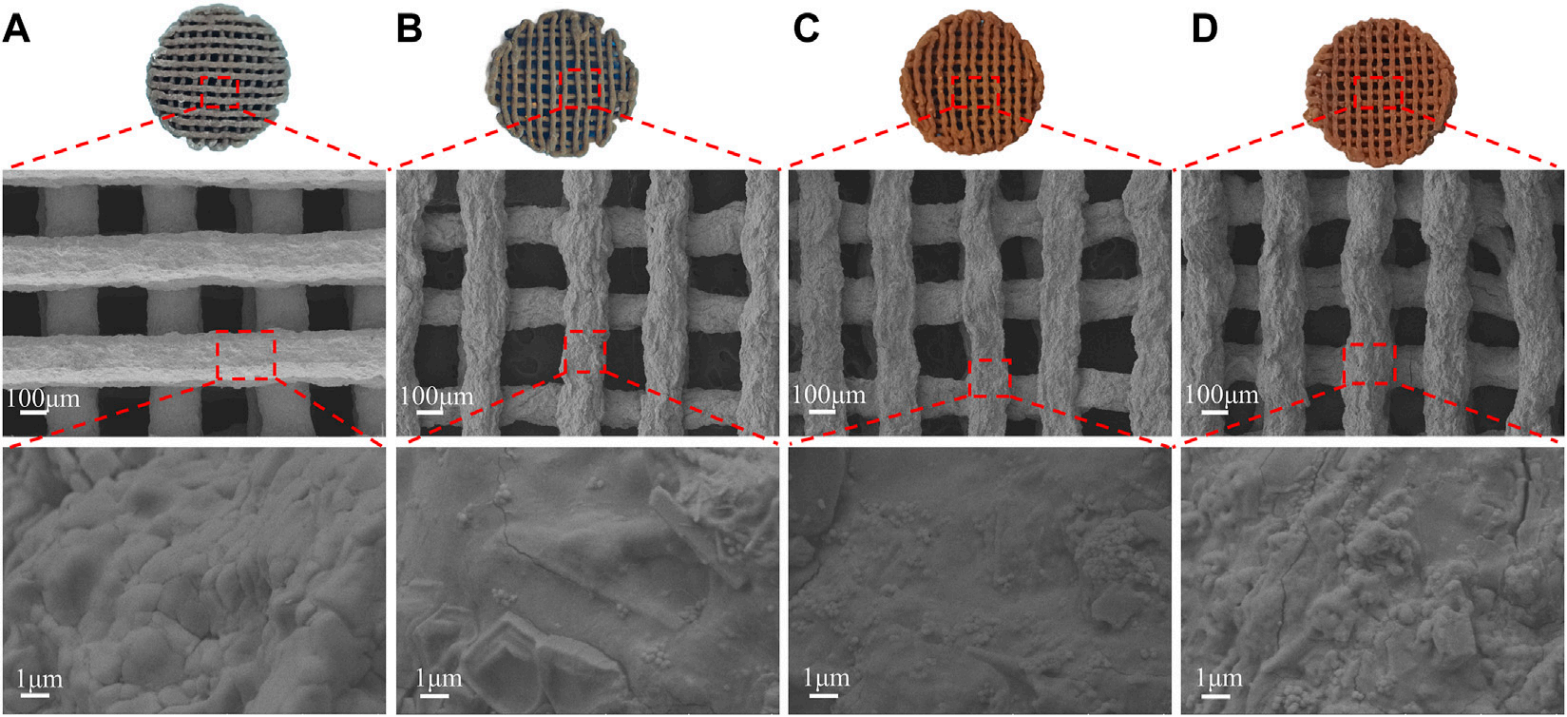
Surface morphology of scaffolds with different DMON@Cur loadings **(A)** 0 wt% **(B)** 3.0 wt% **(C)** 6.0 wt% **(D)** 9.0 wt%.

The SEM (Figure 3) showed the scaffolds doped with DMON@Cur particles of various mass fractions (0,3.0,6.0, and 9.0 wt%) microscopic morphology. All scaffolds had an interconnected porous structure with a rough uncracked surface and pore size of 300 ± 60 μm. The scaffolds became deeper in color as the proportion of Cur-loaded particles increased. The rod-shaped HA was faintly visible and encapsulated inside the scaffolds.

**FIGURE 4:**
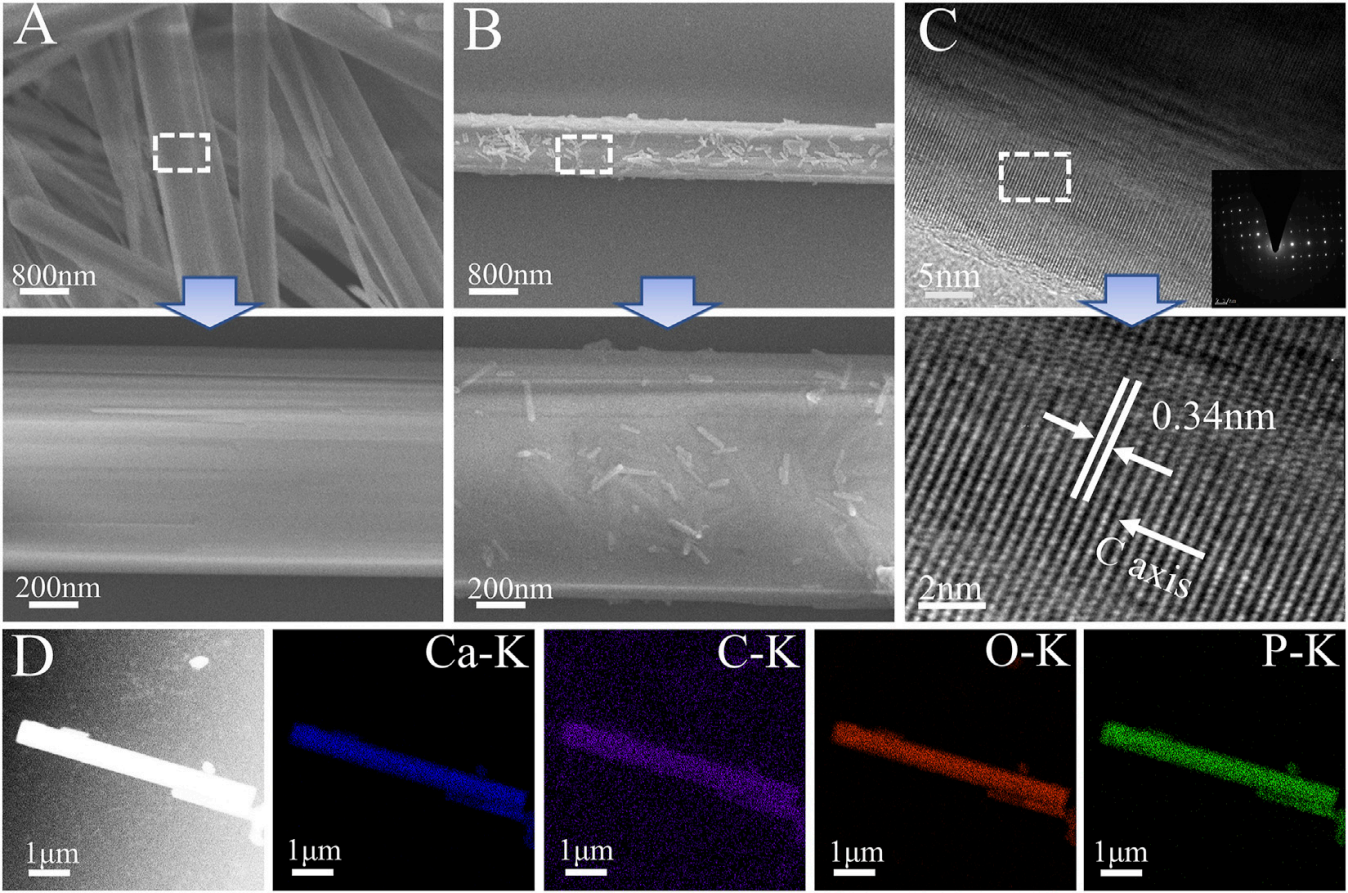
**(A)** SEM micromorphology of HA. **(B)** SEM micromorphology of HA@TA. **(C)** TEM microscopic morphology and electron diffraction of HA. **(D)** Elemental mappings.

The hydroxyapatite(HA) prepared by the hydrothermal method was demonstrated by FESEM (Figure 4A) as homogenous hexagonal long rods (60–170 μm) with a diameter of 818 ± 207 nm and a high aspect ratio (59–278), which was similar to the results of previous studies (Zhang and Darvell, 2010). After TA modification, the surface morphological roughness of HA crystals changed, and some fine HA particles could be seen adsorbed on the surface. It also showed that TA had a high capacity to adsorb and bond to the HA surface (Figure 4B). The HA lattice spacing (Figure 4C) was 0.34 nm, corresponding to the (002) crystal plane of the standard card (JCPDS PDF 9–432), and showed that the HA crystals were developing in a C-axis orientation (Zhang and Zhu, 2005). The elemental distribution of the HA@TA powder was analyzed by EDS (Figure 4D), which showed that the main composition consisted of C(13.87wt%), Ca(29.72wt%), P(18.1wt%), and O(38.31wt%) elements. The presence of C indicated that TA was adsorbed on the surface of HA.

**FIGURE 5:**
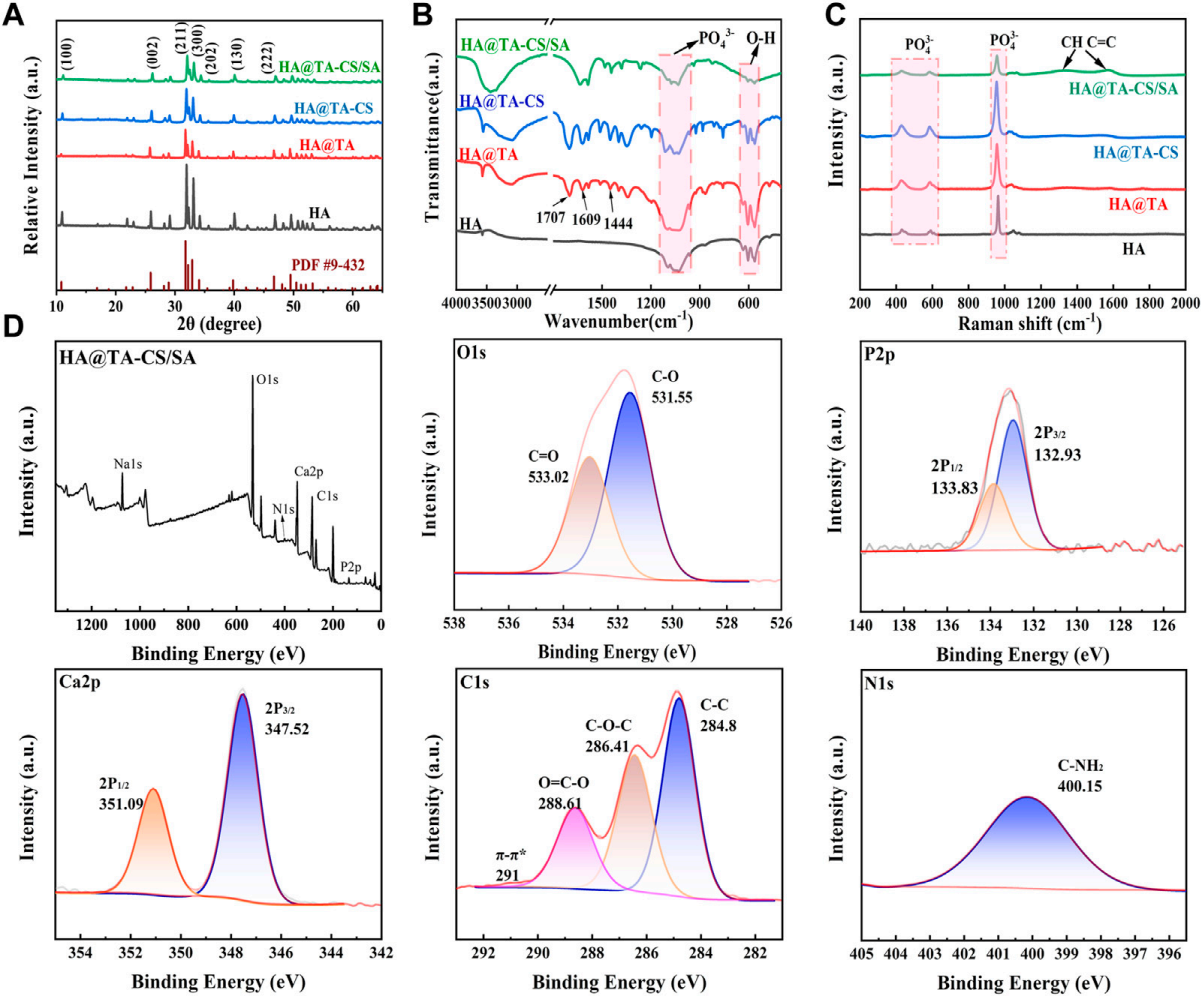
**(A)** XRD spectra of powders. **(B)** FTIR spectra of powders. **(C)** Raman spectra of powders. **(D)** XPS spectra of HA@TA-CS/SA scaffold powders.

The X-ray diffraction (Figure 5A) showed patterns of HA, HA@TA, HA@TA-CS, and HA@TA-CS/SA powders to determine their crystalline properties. The sharp characteristic peaks of HA powders (Figure 5A) were consistent with those of the standard XRD card (JCPDS PDF 9–432) of hydroxyapatite. The characteristic peaks at 10.82°, 21.819°, 25.897°, 28.126°, 28.966°, 31.773°, 32.196°, 32.902°, 39.818°, 46.711°, 49.468° correspond to the planar diffraction peaks of HA (100), (202), (002), (102), (210), (211), (112), (300), (202), (310), (222), (213), where the diffraction peak at (211) crystal plane is the strongest.It indicated that HA powders with high crystallinity were successfully synthesized using the hydrothermal method (Zhang and Zhu, 2005). As previously reported, the most vigorous peak intensity occurred in the (211) lattice plane (Yang et al., 2005). After the surface modification, however, the intensity diffraction peak of HA powders was decreased. Moreover, the functional groups of the material and their possible interactions within the scaffold were assessed using FTIR and Raman techniques. The FTIR spectra (Figure 5B) showed the HA powder and after organic modified HA powder. The characteristic peaks at 1093, 1034, 602, and 564 cm^-1^ were PO_4_^3-^ and the absorption peaks at 3571 and 633 cm^-1^ were attributed to hydroxyl groups. These main vibrational peaks were typical of the characteristic peaks of HA (Koutsopoulos, 2002; Zhang and Darvell, 2010). Compared to the pure HA powder, the HA@TA powder had C-O stretching peak (1707 cm^−1^), C-O stretching peak (1609 cm^−1^), C-C-C aromatic ring stretching peak (1444 cm^−1^), and C-C bond corresponding to the aromatic ring 754 cm^−1^ peak(Hussain et al., 2022). The absorption peak at 1552 cm^−1^ of the HA@TA-CS powder spectrum corresponded to the -NH_2_ bending vibration. The absorption peak (1440 cm^−1^) was related to the electrostatic interaction between the NH^3+^ group of CS and the phenoxy (Ph-O-) group of HA@TA(Lee et al., 2023). The absorption peaks of the HA@TA-CS/SA powder were C-O-C stretching at 1030 cm^−1^ and OH- stretching at 3450 cm^−1^. The observed bands at 1625 and 1420 cm^−1^ for SA were attributed to carboxyl groups and asymmetric and symmetric COO- stretching vibrations, respectively. The antisymmetric stretching of COO- in SA shifts towards the lower band, and wave numbers overlap the antisymmetric stretching peaks of COO- at 1546 cm^−1^ and 1595 cm^−1^. It confirmed the presence of ionic interactions between cationic CS and anionic SA via electrostatic interactions (Ke et al., 2010). The Raman spectra (Figure 5C) also showed the typical characteristic peaks of HA, with the modified powder having CH- and C=C double bonded peaks at 1350 cm^−1^ and 1550 cm^−1^, respectively. The most pronounced peaks on the surface of the scaffold demonstrated successful modification on the surface of HA. Based on the XPS spectra of HA@TA-CS/SA scaffold powders results (Figure 5D), the deconvolution peaks of Ca2p and P2p were typical of the elements and families of HA crystals (Yang et al., 2005). The deconvolution peaks of C-C (284.8 eV), C-O-C (286.41 eV), O=C-O (288.61 eV), and π-π* (291.0 eV) in the C1s spectrum and C=O (533.02 eV) and C-O (531.55 eV) in the O1s spectrum reflected organic bonding on the HA ceramic surfaces (Hussain et al., 2022; Lee et al., 2023). The appearance of C-NH2 (400.15 eV) in the N1s spectrum also demonstrated the introduction of CS in the scaffold (Lee et al., 2023).

**FIGURE 6:**
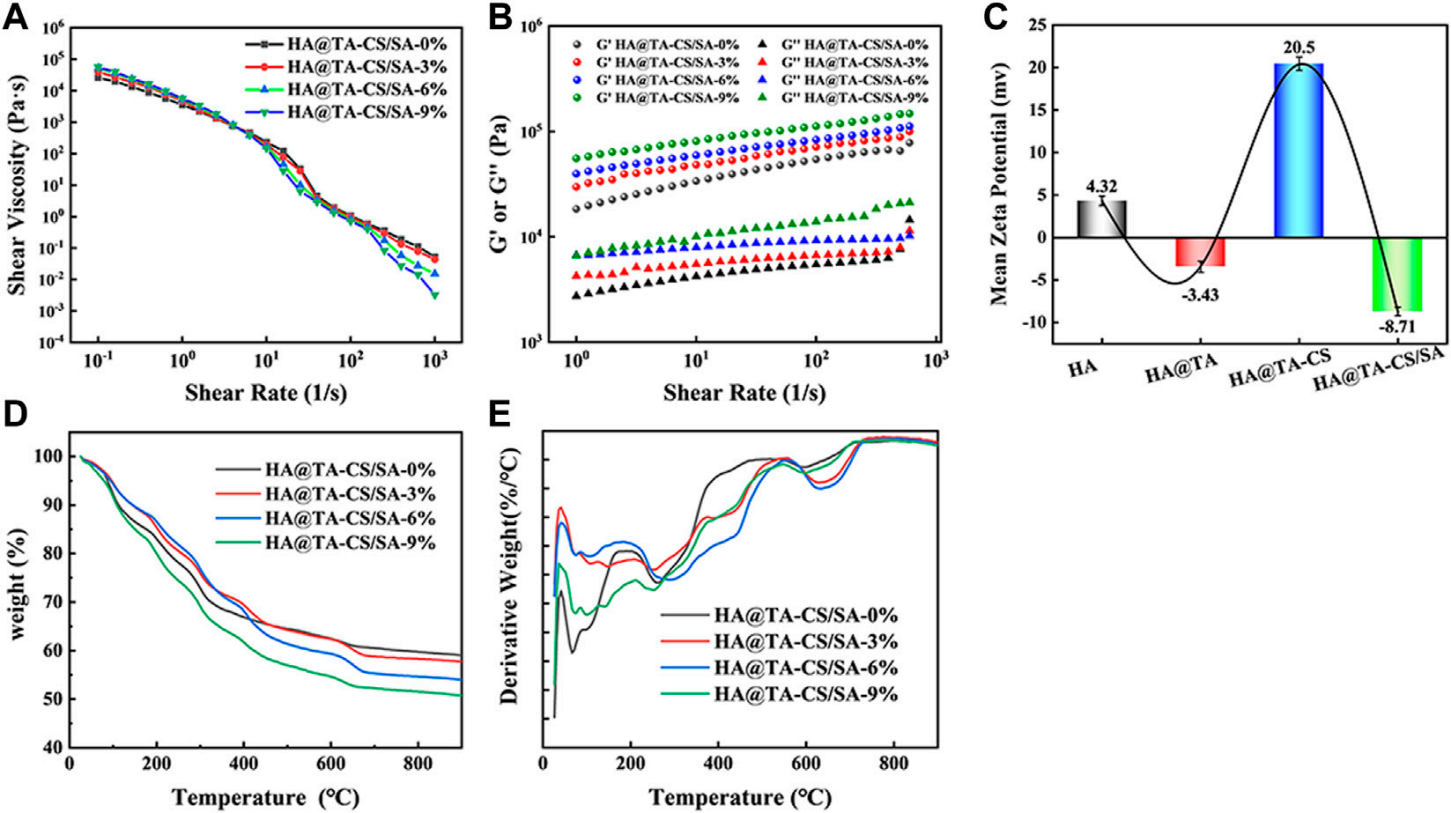
**(A)** Variation curve of viscosity with the shear rate for curcumin-loaded HA@TA-CS/SA scaffolds. **(B)** Variation curves of G′ and G″ with the shear rate for curcumin-loaded HA@TA-CS/SA scaffolds. **(C)** zeta potential **(D)** TG curves of curcumin-loaded scaffolds **(E)** DTG curves of curcumin-loaded scaffolds.

Rheological performance is an essential judgment in examining the printable properties of the slurry. After adding DMON@Cur particles with mass fractions of 0, 3.0, 6.0, 9.0 wt% to the HA@TA-CS/SA slurry, the pastes' rheological characteristics (Figure 6A) were examined. The apparent viscosity of all slurry decreased significantly with increasing shear rate showing an apparent “shear thinning” phenomenon typical of pseudoplastic fluids. In addition, the higher the mass fraction of DMON@Cur particles added, the apparent viscosity of the slurry was slightly reduced, but the effect was not significant. Frequency scans (Figure 6B) of the slurries showed that the G′ values for all slurries remained essentially constant throughout the frequency range, indicating good crosslinking (Dong et al., 2022). The addition of DMON@Cur carrier particles had little effect on the internal crosslinking mechanism of the slurries, which were stable and continuous.

We performed zeta potential tests during each step of the scaffold preparation to determine the binding pattern within the material (Figure 6C). At the initial stage, the synthesized HA surface displayed a positive charge of 4.32 mv. After the TA modification, the material exhibited a negative charge of −3.43 mv (Hussain et al., 2022). Following the introduction of CS, the material displayed another positive charge of 20.5 mv. Finally, after the binding of SA, the material displayed a negative charge of −8.71 mv. It had been reported that TA could easily adhere to the HA surface mainly through hydrogen bonding. Based on the combined results of XRD, FT-IR, Raman, and XPS analysis, we speculated that the catechol group of TA formed a hydrogen bond with the hydroxyl group of HA and wrapped tightly on the surface of HA to form a TA film. At this time, the HA@TA particle had a negative charge. After cationic CS was introduced, the NH^3+^ group in CS adsorbed on the surface of HA@TA particles through electrostatic interaction (Shu et al., 2001). By stirring and electrostatic mutual repulsion, the charged particles could be uniformly dispersed in the slurry. After adding SA, all positively charged HA@TA-CS particles were attracted to each other by electrostatic interaction with the negative charge of SA, and finally, the slurry was formed. The alternating positive and negative changes in Zeta potential with each step of material modification proved that the material components were bonded through hydrogen bonding and electrostatic interactions.

Figures 6D, E showed the TG and DTG curves of the scaffolds loaded with different mass fractions (0, 3, 6, 9 wt%) of DMON@Cur nanoparticles. The weightlessness process of all scaffolds went through roughly three stages. The first stage was roughly 40°C–150°C due to the evaporation of free water in the scaffold. The main thermal decomposition stage occurred at 200°C–700°C. This stage saw the decomposition of polymer tannins, chitosan, sodium alginate, and curcumin. The weight loss of the scaffold gradually stabilized in the third stage 700°C–900°C, and the hydroxyapatite in the scaffold underwent a slow phase transition. All scaffolds' final weight loss ratio was positively correlated with the mass of loaded curcumin.

### 3.3 Compressive strength of scaffolds

**FIGURE 7:**
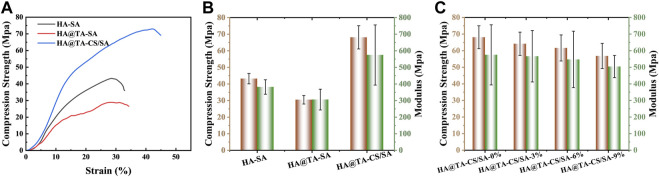
**(A)** The presentative compressive stress-strain curves of the HA-SA, HA@TA-SA, and HA@TA-CS/SA scaffolds. **(B)** compressive stress and Young’s modulus of the HA-SA, HA@TA-SA, and HA@TA-CS/SA scaffolds. **(C)** compressive stress and Young’s modulus of the DMON@Cur loaded scaffolds.

The compressive stress-strain curves of the HA-SA, HA@TA-SA, and HA@TA-CS/SA scaffolds are shown in Figure 7A. The strains were in the range of 0%–45% for all three groups of scaffolds. It could be seen that the compressive strength of the HA-SA scaffold without TA and CS modification was 43.23 Mpa. When TA and CS modified the HA-SA scaffold, the prepared HA@TA-CS/SA scaffold had the highest compressive strength increasing to 68.09 Mpa. The prepared scaffolds were still competitive in terms of mechanical properties when compared with published studies (Chi et al., 2022; Rezania et al., 2022; Zhao et al., 2022). To confirm the great increase of compressive strength for the HA@TA-CS/SA scaffold was due to the solid electrostatic interactions by CS, the HA@TA-SA scaffold was designed and produced without CS modification. It is notable that the HA@TA-SAC scaffold, however, was less intense than the HA-SA scaffold, being the least strong of the three groups at 30.47 Mpa. In addition, the HA@TA-CS/SA scaffold had the highest maximum strain of the three groups at 43%. At the yield stress point of the other two groups of scaffolds, the compressive strength of the prepared HA@TA-CS/SA scaffold for the same period was 65 Mpa.

The three groups of scaffolds compared Young’s modulus (Figure 7B), with the HA@TA-CS/SA scaffold still having the best modulus (574.92 Mpa), which was higher than the HA-SA scaffold without CS and TA modifications (381.75 Mpa) by 50.6%. As a comparison, Young’s modulus of the HA@TA-SA scaffold (305.99 Mpa) decreased by 46.77%. Compared the results with the Zeta potential data (Figure 6C), the HA@TA was negatively charged and repelled the negative charge of SA, which was the main reason for the deterioration in mechanical strength. After introducing positive charged CS as a bridge between the TA and SA, part of the TA’s hydroxyl group made hydrogen bonds with the CS, as indicated in the IR spectrum. While the other portion protonates the -NH_2_ group of the CS, forming an electrostatic connection with the TA’s phenoxy group (Ph-O-). Particles with a positively charged outer layer were generated during the slurry preparation process and then added to the anionic SA solution.

The CS in the outer layer could create hydrogen and amide bonds with the SA, as revealed by the XPS spectra (Figure5D). The anionic SA and cationic CS rely on electrostatic interactions for homogenous binding. At last, Ca^2+^ ions chelated the SA in the slurry to generate a double network structure maximized scaffold strength (Song et al., 2022). The compressive strength and Young’s modulus of scaffolds (Figure 7C) with the addition of different mass fractions of particles showed that both the compressive strength and Young’s modulus of the scaffolds were decreased slightly. Owing to their stiffness differences, microspheres produce stress concentrations in materials, making them more brittle. The microspheres may also propagate defects and cracks in the material, leading to the fragmentation of the cross-linkage network between the internal components of the scaffolds, which weakens their mechanical properties.

The mechanical properties of bone repair scaffolds have an important role in bone tissue regeneration. Especially in the initial stage of tissue regeneration, it can provide the necessary mechanical support and sufficient stability for the bone repair site, so that the conditions for tissue growth and nutrient delivery exchange during bone tissue regeneration can be created. Secondly, the high modulus bone repair scaffold can simulate the mechanical properties of normal bone, reduce the displacement and stress concentration during bone reconstruction, promote the growth and regeneration of bone cells, and facilitate bone repair (Zhang et al., 2021a; Zhang et al., 2022b).

### 3.4 *In vitro* degradation and mineralization

**FIGURE 8:**
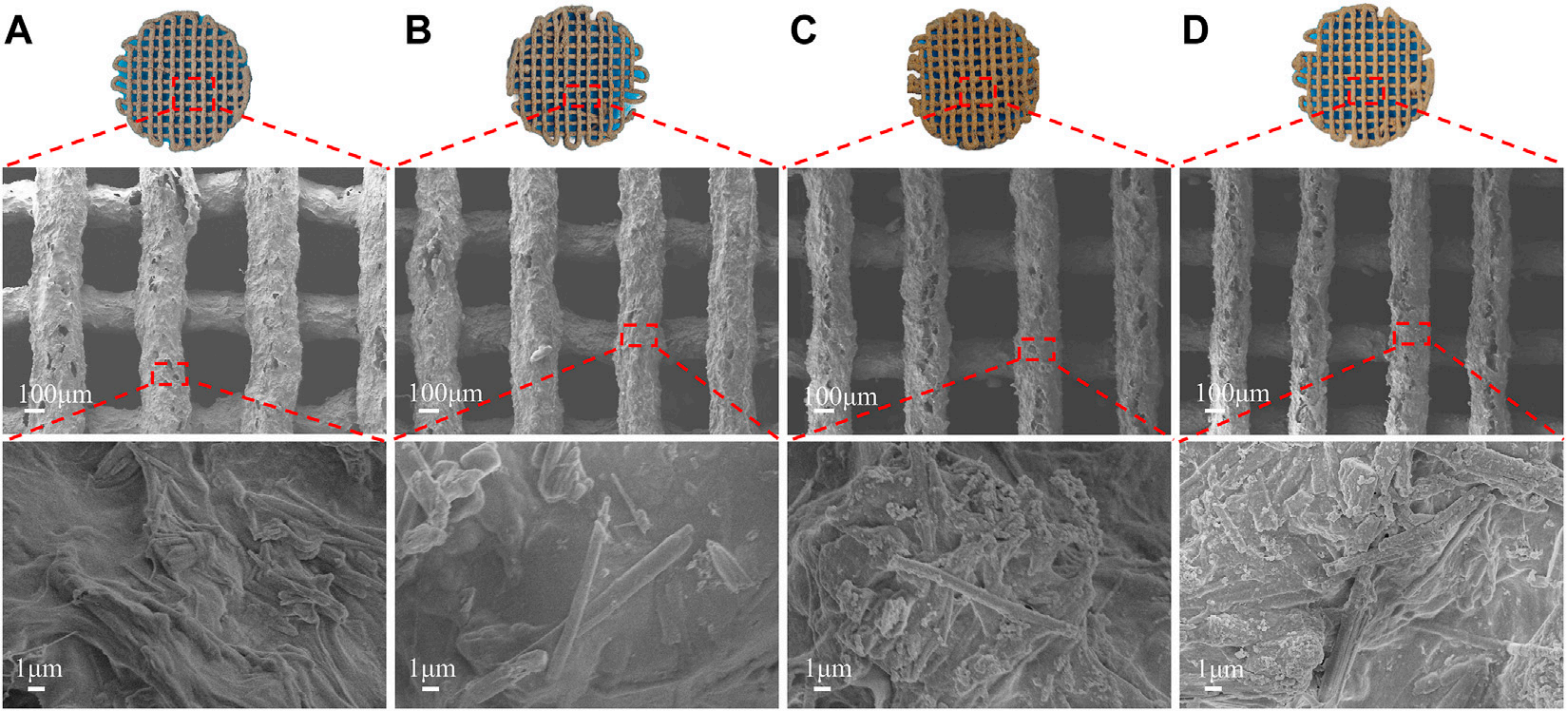
Surface morphology of the DMON@Cur loaded scaffolds after 14 days of *in vitro* degradation. **(A)** 0 wt%. **(B)** 3 wt%. **(C)** 6 wt%. **(D)** 9 wt%.

The rate of deterioration *in vitro* of bone tissue engineering scaffolds is an essential characteristic. The optimal pace of scaffold breakdown should be proportional to the rate of new bone formation. It is also vital to research the long-term behaviour of the scaffold in the same physiological context for medication application. Following 14 days in PBS media, (Figure 8),optical photos of the dried scaffolds revealed that all scaffolds retained their structure and that additional fractures were discovered on the surface of the scaffolds, but no substantial deformation occurred. More rod-shaped HA and DMON@Cur particles were observed on the surface of the scaffolds when the microstructure was examined using SEM (Figure 8). The number of particles exposed exhibited a similar pattern to the amount of medication loaded. Figure 10A depicted the DMON@Cur-loaded scaffolds' weight degradation curves, revealing that all scaffolds degraded similarly, with an overall sluggish, fast, and slow degradation rate. The greatest rate of deterioration occurred on day 7, followed by a slowing on day 12. The total mass on day 14 decreased to 23.47, 21.85, 21.56, and 19.38%, respectively, with small variances in degradation mass closely proportional to the drug-loaded mass.

**FIGURE 9:**
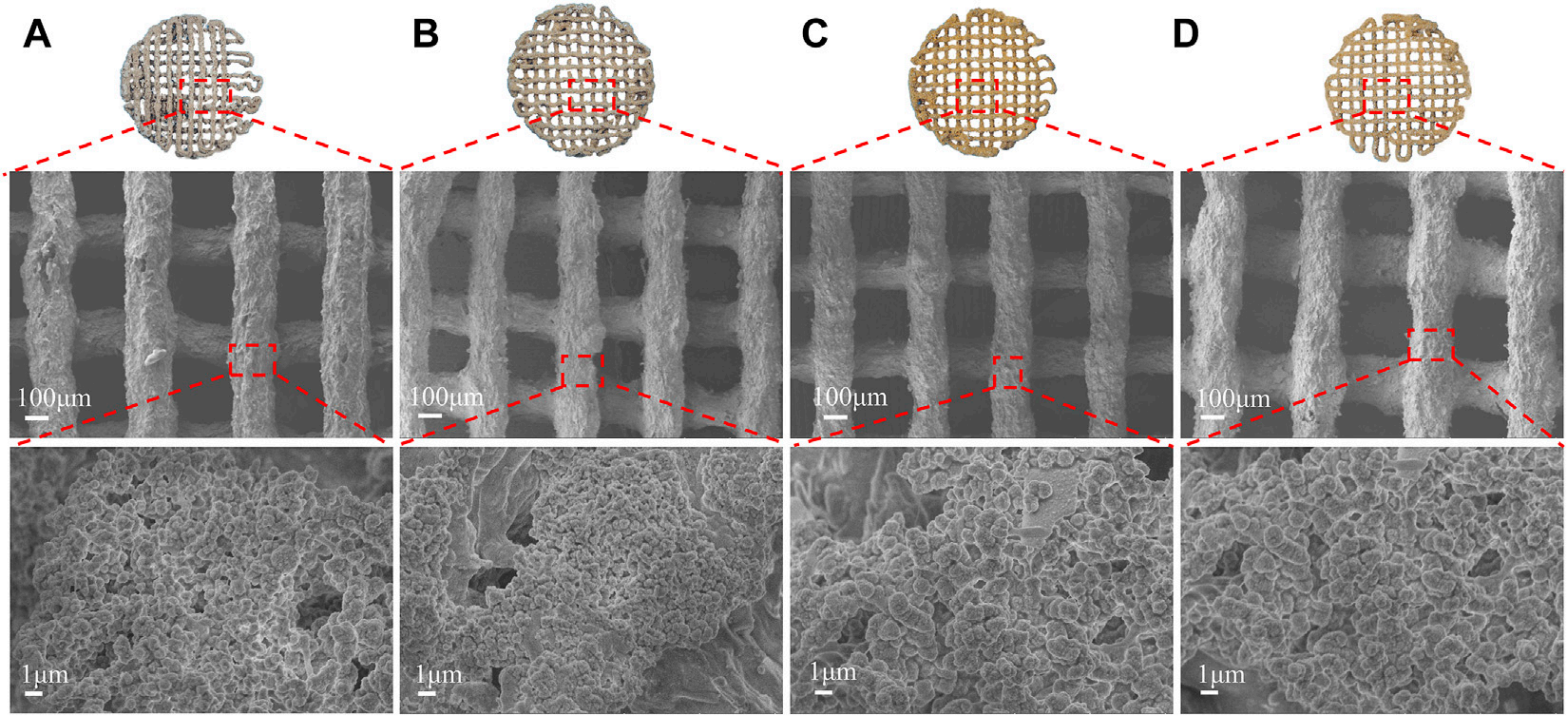
Surface morphology of scaffolds with different drug loadings after 14 days of immersion in SBF. **(A)** 0 wt% **(B)** 3 wt% **(C)** 6 wt% **(D)** 9 wt%.

It is used to measure the biological activity of osteogenesis *in vitro* that the capacity to generate apatite on the surface of biological materials in simulated body fluids (SBF) (Chen et al., 2020). Figure 9 depicted optical and SEM photos of the scaffold after 14 days of immersion in SBF. The optical pictures revealed that the structure suffered no significant damage due to the immersion. Ca/P or hydroxyapatite deposits appeared on the surface, and the morphology was intact. SEM revealed similarly dispersed white spherical particles of identical size on the surface of all scaffolds (Olad et al., 2019; Chen et al., 2020). There were no significant differences between the four scaffold groups. The presence of calcium and phosphorus on the surface of the scaffolds was confirmed by EDS elemental analysis of the white spherical particles detected. Calcium cations have been found to play a vital function in the mineralization of scaffolds as crosslinking agents in scaffold preparation (Song et al., 2022). Moreover, the amino group of CS may enhance the deposition of biominerals (Xu and Liang, 2022; Hakimi et al., 2023). Consistent with our findings, all DMON@Cur-loaded scaffolds exhibited excellent mineralization virtually uniformly, indicating the scaffolds' potential capacity to promote new bone formation.

### 3.5 *In vitro* release of curcumin

**FIGURE 10:**
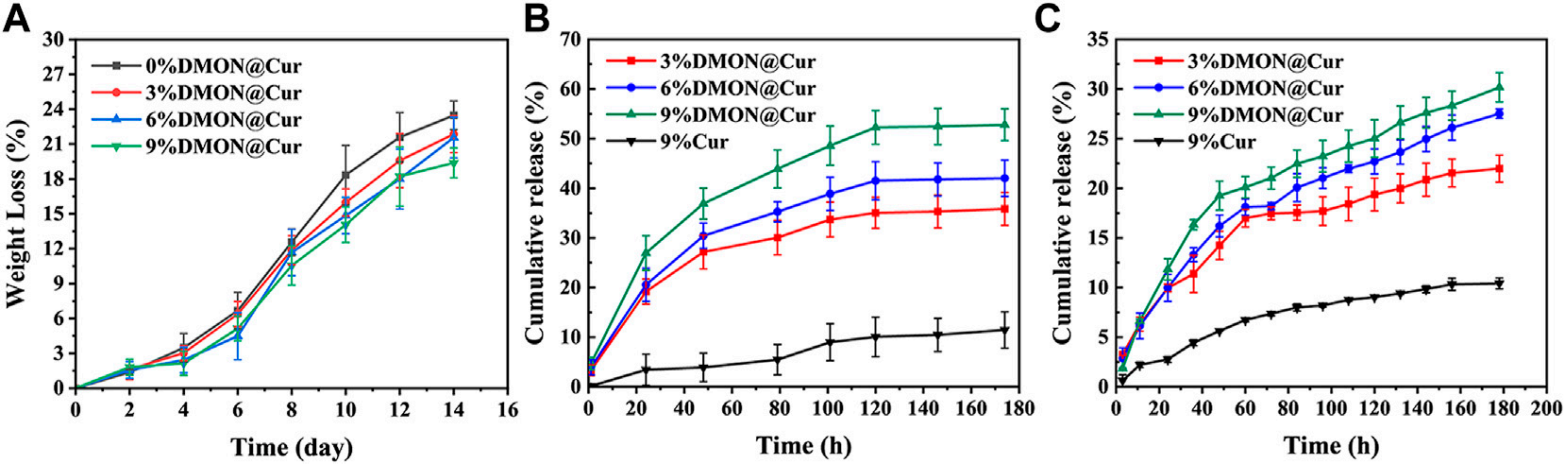
**(A)**The degradation rate of curcumin-loaded scaffolds. **(B)** Drug release from curcumin-loaded scaffolds at pH7.4 **(C)** Drug release from curcumin-loaded scaffolds at pH5.3.

Curcumin is often used to prevent and treat various diseases and has various pharmacological properties, including anticancer, anti-osteoarthritis, antioxidant, and antibacterial. Nevertheless, its insolubility in water (only 0.4 ug/ml) and rapid degradability at physiological pH severely reduce its bioavailability (Hamilton and Gilbert, 2023; Jiang et al., 2023). As a result, this investigation employed curcumin as a typical drug template being loaded into the scaffold to explore the scaffolds' *in vitro* drug release at pH 7.4 and pH 5.3. As shown in Figure 10B, the cumulative curcumin release rates of the DMON@Cur-loaded (3.0,6.0, and 9.0 wt%) scaffolds at pH 7.4 over 180 h were 52.78% ± 3.2%, 42.02% ± 3.67%, and 35.84% ± 3.32%, respectively. Also at pH 5.3, the release rates of cumulative curcumin over 180 h were 21.98% ± 1.35%, 27.51% ± 0.46%, and 30.16% ± 1.46% (Figure 10C). As a comparison, the cumulative release rate of the scaffolds directly doped with 9 wt% curcumin was only 11.45% and 10.41% during the same period. The release of curcumin-loaded scaffolds was slower and more sustained at the pH of the inflammatory environment. It showed that the scaffolds loaded with DMON@Cur particles exhibited better drug release performance for all gradient concentrations than the control group. There was an overall rapid release trend at pH 7.4, followed by a gradual smoothing out, reaching the maximum drug release concentration at 120 h. In contrast, at pH 5.3, the release pattern of curcumin was first rapid and then slow, with an inflection point at 60 h. The scaffolds loaded with a more mass fraction of DMON@Cur particles simultaneously had greater drug release concentrations.

It is presumably because more DMON@Cur particles were exposed on the surface as the scaffolds continued to degrade in PBS, leading to more drug release. Acidic inflammatory environments protonate chitosan in the scaffold and form more amide bonds with sodium alginate, leading to tighter binding, slower degradation and more sustained drug release during scaffold degradation. The composite of curcumin with mesoporous material might restrict curcumin in monomorphic form within the spaces of a porous material (Ananth et al., 2022). In this way, the solubility and bioavailability of curcumin could be significantly improved.

### 3.6 Biocompatibility assay

**FIGURE 11:**
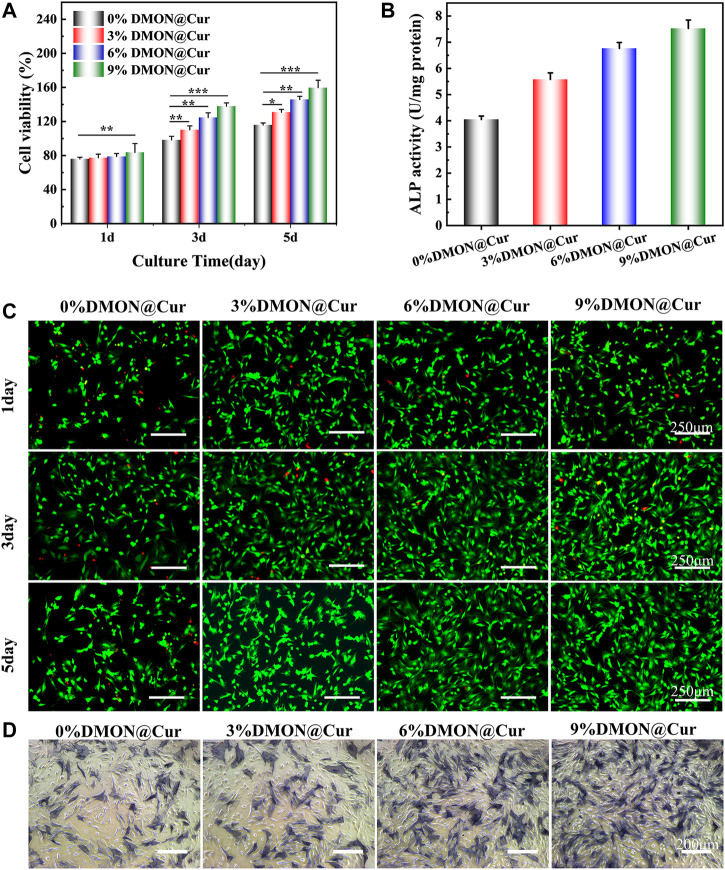
**(A)** CCK-8 assay for scaffold cytotoxicity. **(B)** ALP quantitative analysis **(C)** Live-dead staining of cells **(D)** ALP osteogenic differentiation staining.

*In vitro* cell assay is an essential tool to detect growth inhibition, functional changes, cell lysis, death, or other toxic reactions of cells after exposure to biological materials in an isolated environment that simulates the growth environment of an organism (Ananth et al., 2022; Claro et al., 2023). The cell viability of BMSCs cultured in DMON@Cur-loaded HA@TA-CS/SA scaffold extracts assayed by the CCK-8 method is demonstrated in Figure 11A. The results showed that all scaffolds were non-toxic. There was no significant difference between the groups on the first day. Still, with time, the cell proliferation of the DMON@Cur-loaded scaffolds was significantly better than that of the positive control group. The cell survival rate reflected an increasing trend, and the proliferation was positively correlated with the DMON@Cur loading, showing good biocompatibility. Figure 11C showed the results of BMSCs cells stained under a fluorescence microscope, and the overall situation is consistent with the detection of the CCK-8 assay. The cell morphology of the DMON@Cur-loaded scaffolds was more spreading and numerous, which strongly confirmed that the scaffold had good biocompatibility and could promote the proliferation of BMSCs cells (Zhang et al., 2021b). The synthesized scaffold materials using natural polymers have shown very good biocompatibility. Figures 11B, D showed the quantitative analysis and staining results of alkaline phosphatase (ALP) after BMSCs cells were cultured for 7 days in the infiltrate of HA@TA-CS/SA-loaded curcumin scaffold. The results showed that the scaffold with more drug loading had a deeper and denser ALP staining status, indicating that the curcumin-loaded scaffold could significantly promote the differentiation of BMSCs cells (Li and Zhang, 2018; Chen et al., 2021; Inchingolo et al., 2022).

On the other hand, curcumin could promote the survival and proliferation of osteoblasts by eliminating the inhibitory effect of reactive oxygen species on the Nrf2 signaling pathway to reduce osteoblast apoptosis and maintain their differentiation function, as reported by Li et al. (Wu et al., 2019; Dang et al., 2021). In conclusion, all curcumin-loaded scaffolds have good proliferation-promoting and differentiation effects on BMSCs cells, showing their great potential in bone repair properties.

### 3.7 Antibacterial performance

**FIGURE 12:**
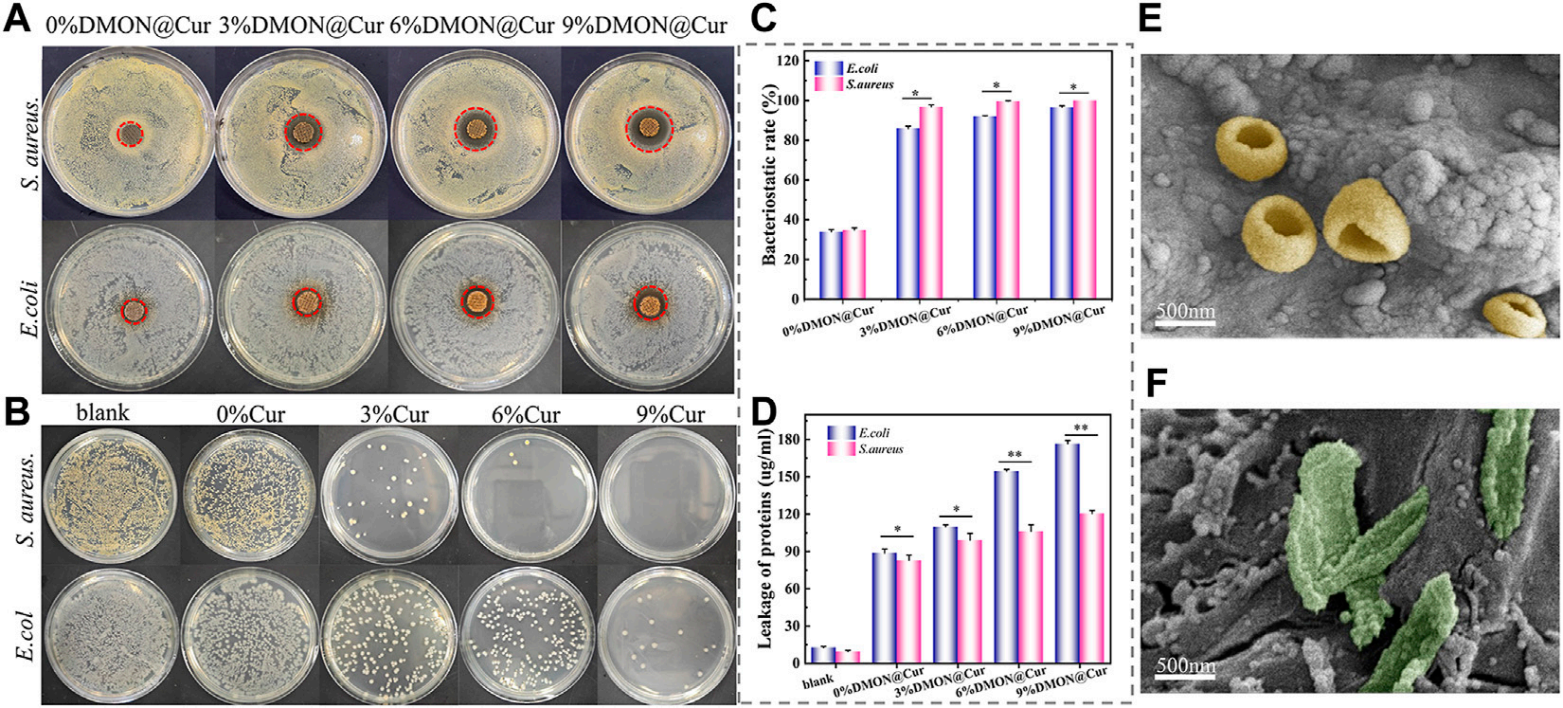
**(A)** and **(B)** Antibacterial capacity of curcumin-loaded scaffolds for testing *S.aureus* and **(E)** *coli* by disk diffusion and plate coating methods. **(C)** BCA kit detects protein concentrations released by bacteria co-cultured with curcumin-loaded scaffolds. **(D)** bactericidal rate of *S.aureus* and **(E)** *coli* after treatment with the curcumin-loaded scaffolds. **(E, F)** SEM after co-cultivation of *S.aureus* and **(E)** *coli* with curcumin-loaded scaffolds for 24 h.

Selected representative *S.aureus* and *E.coli* characterize the antibacterial activity of the scaffolds, qualitatively and quantitatively, by using the disk diffusion method and plate coating method, respectively. As shown in Figure 12A, the inhibition ring diameters of the scaffolds were 15.4 ± 0.6 mm, 19.3 ± 0.8 mm, 23.6 ± 0.5 mm, and 27.4 ± 0.8 mm after 24 h incubation with DMON@Cur-loaded scaffolds (0, 3,6, and 9 wt%) in *S.aureus* medium. The inhibition ring diameters were 15.8 ± 0.3, 16.4 ± 0.2, 17.2 ± 0.3 and 18.0 ± 0.2 mm, respectively, after 24 h incubation with the same concentration gradient of DMON@Cur-loaded scaffolds in *E.coli* culture dishes. All concentration gradients of DMON@Cur-loaded scaffolds showed inhibition, and the inhibition effect was significantly stronger for *S.aureus* than for *E.coli*. Adding 0% DMON@Cur-loaded particles to the scaffolds also produced a small amount of inhibition, attributed to the antimicrobial properties of TA and CS in the scaffolds. It is noteworthy that the TA and CS added to the scaffold were only in small amounts, and the bacterial inhibitory effect of the scaffold increased significantly with the increase of the curcumin-loaded drug content, which was mainly influenced by curcumin. Figure 12B showed the results of the DMON@Cur-loaded scaffold co-cultured with *S.aureus* and *E.coli* bacteria for 24 h after plate coating. It coincided with the results of the inhibition test; Figure 12C showed the bactericidal rate of *S.aureus* and *E.coli* after treatment with the DMON@Cur-loaded HA@TA-CS/SA scaffolds. Adding 9 wt% of DMON@Cur-loaded microspheres to the scaffold produced 99.99% and 96.56% bactericidal effects against *S.aureus* and *E.coli*, respectively. The above test proved that the DMON@Cur-loaded HA@TA-CS/SA scaffolds had a good antibacterial effect, especially on *S.aureus* bacteria, which was also in line with the results of previous studies (Doustdar et al., 2022).

The bacteria morphology was observed under SEM after co-cultivation of *S.aureus* and *E.coli* with DMON@Cur-loaded scaffolds for 24 h, respectively (Figures 12E, F). The rough surface of the scaffolds was favourable for bacterial adhesion. It was also observed that the cell membrane of *S.aureus* and *E.coli* were broken to expose the intracellular material, and DMON@Cur-loaded microspheres were distributed around them. In contrast, most *S.aureus* had more intact cell structures than *E.coli*, and more folds could be seen on their cell surfaces. It is theorized that curcumin disrupted bacteria’s cell membrane structure, ultimately resulting in their demise. Despite the qualitative results observed for visualization in electron micrographs, it had been shown that the intracellular leaked-out proteins could be quantitatively characterized using the BCA protein kit. As shown in Figure 12D, according to the protein assay, both the *E.coli* groups and the *S.aureus* groups were able to cause a large amount of intracellular protein leakage after co-culture with the DMON@Cur-loaded scaffolds compared to the blank groups. Among them, the curcumin-loaded scaffolds containing 9 wt% of DMON@Cur particles were co-cultured with *Escherichia coli* and *Staphylococcus aureus* with protein concentrations up to 176.59 ± 2.6 and 120.54 ± 2.53 μg/mL, respectively. It is consistent with the results visualized in the electron micrographs, indicating that our hypothesized mechanism of bacterial inhibition of the DMON@Cur-loaded scaffold was bio-compatible. In addition, the *S.aureus* group shed less intracellular material. That could be because the thick layer of peptidoglycan on the outside of Gram-positive bacteria acts as a barrier to releasing intracellular chemicals. Gram-negative bacteria lack such a robust protective barrier, causing *E.coli* to break down and spill out vast amounts of intracellular protein (Yuan et al., 2022).

## 4 Conclusion

In summary, we prepared a series of HA@TA-CS/SA scaffolds with different curcumin-loaded silica microspheres (0,3.0,6.0, and 9.0 wt%) using 3D printing. XRD and TEM showed that the synthesized long rod-shaped hydroxyapatite grew directionally along the C-axis with a high aspect ratio. IR, Raman, XPS, and zeta potential showed that the components within the scaffold were bound to each other in layers by hydrogen bonding and solid electrostatic interactions. The rheological test revealed that the slurry was a pseudoplastic fluid with a stable bond. Their rheological properties were unaffected by the addition of silica-loaded particles. The scaffolds also had good mechanical qualities (compressive strength 68.09 Mpa). In addition, SEM and EDX demonstrated that the scaffolds had high biomineralization capability and a good degradation rate. The synthesized scaffold’s have high cytocompatibility which can boost the proliferation of BMSCs significantly. Curcumin’s solubility and bioavailability were enhanced by the scaffold, as demonstrated by *in vitro* drug release test. Moreover, the HA@TA-CS/SA-9% DMON@Cur scaffolds inhibited *S.aureus* and *E.coli* with 99.99% and 96.56% effectiveness, respectively. The scaffolds' antibacterial activity mechanism was confirmed using SEM, and the BCA protein assay, which was curcumin disrupts bacteria’s cell membrane structure. Overall, the results indicated that HA@TA-CS/SA-9% DMON@Cur scaffolds had considerable potential for therapeutic bone tissue engineering.

## Data Availability

The original contributions presented in the study are included in the article/supplementary material, further inquiries can be directed to the corresponding author.
